# Monoamine oxidase A upregulated by chronic intermittent hypoxia activates indoleamine 2,3-dioxygenase and neurodegeneration

**DOI:** 10.1371/journal.pone.0177940

**Published:** 2017-06-09

**Authors:** Chun-Sing Lam, Jing-Jie Li, George Lim Tipoe, Moussa B. H. Youdim, Man-Lung Fung

**Affiliations:** 1 School of Biomedical Sciences, The University of Hong Kong, Hong Kong, China; 2 Research Centre of Heart, Brain, Hormone & Healthy Aging, Li Ka Shing Faculty of Medicine, The University of Hong Kong, Hong Kong, China; 3 Eve Topf Center for Neurodegenerative Diseases Research, Faculty of Medicine, Technion-Israel Institute of Technology, Haifa, Israel; Indian Institute of Integrative Medicine CSIR, INDIA

## Abstract

Co-morbid depression is prevalent in patients with obstructive sleep apnea. Here we report that monoamine oxidase A (MAO-A) plays pathogenic roles in the comorbidity. We found that chronic intermittent hypoxia significantly increased the MAO-A expression in the rat hippocampus and markedly decreased the dendritic length and spine density in the pyramidal neurons with less pre- and post-synaptic proteins. The MAO-A upregulation resulted in increased 5-hydroxyindoleacetic acid/serotonin ratio, oxidative stress, leading to NF-κB activation, inflammation and apoptosis. Also, the expression of cytokine-responsive indoleamine 2,3-dioxygenase-1 (IDO-1) was significantly augmented in hypoxia, resulting in increased kynurenine/tryptophan ratio and lowered serotonin level in the hippocampus. Moreover, depressive-like behaviors were observed in the hypoxic rat. Administration of M30, a brain-selective MAO-A inhibitor with iron-chelating properties, prior to hypoxic treatment prevented the aberrant changes in the hippocampus and depressive behavior. In human SH-SY5Y cells expressing MAO-A but not MAO-B, hypoxia significantly increased the MAO-A expression, which was blocked by M30 or clorgyline. Collectively, the MAO-A upregulation induced by chronic intermittent hypoxia plays significant pathogenic role in oxidative stress, inflammation and IDO-1 activation resulting in serotonin depletion and neurodegeneration.

## Introduction

Obstructive sleep apnea (OSA) is a major type of sleep-disordered breathing prevalent in 2–7% of adults globally [1]. Co-morbid depression is common (21–41%) in OSA patients [2–4]. Recent studies showed that symptoms of depression were alleviated in OSA patients treated with continuous positive airway pressure [5, 6]. Besides, depressive-like behavior was observed in experimental animals given the treatment of chronic intermittent hypoxic (CIH) [7, 8]. These studies suggest causality between OSA and depression, but there is a paucity of mechanistic delineation of the pathophysiological link of the comorbidity.

Brain monoamine oxidase A (MAO-A) plays an important role in maintaining the availability of monoamine neurotransmitters [9]. Dysregulated MAO-A activities significantly alter the homeostatic balance of monoamines that underpin pathogenesis of depression. In fact, overactivation of MAO-A has been reported in the brain of clinically depressed patients and in the postmortem brain [10, 11]. Also, neurodegeneration induced by elevated MAO-A activities was associated with depressive behavior in rodents with chronic stress [12].

Although the role of inflammation in depression is highly contested, inflammation was reportedly observed in the brain of clinically depressed patients [13]. Inflammatory cytokine-responsive indoleamine-2,3-dioxygenase-1 (IDO-1) activation plays an important pathogenic role in the development of depressive-like behavior in experimental animals [14, 15]. IDO-1 catalyzes the first, rate-limiting step, in the tryptophan catabolism pathway, generating kynurenine and resulting in reduced levels of serotonin. Additionally, it has been demonstrated that a metabolite of the kynurenine pathway, quinolinic acid, can be neurotoxic. In fact, neurotoxic metabolites upon IDO-1 activation were reportedly to induce neurodegeneration [16, 17]. Here we examined the hypothesis that MAO-A upregulation induced by chronic intermittent hypoxia causes inflammation and IDO-1 activation, which significantly contribute to the serotonin deficiency and neurodegeneration.

Brain permeable M30, 5[-N-Methyl-N-propargylaminomethyl]-8- hydroxyquinoline), is a synthetic compound composed of propargyl moiety and prototype of iron-chelator VK28 [18]. Thus, M30 possesses chemical properties of brain-selective MAO inhibitors and iron-chelating free radical scavengers [19]. These properties have been shown to be central to the protective effect of M30 against the pathogenic processes of neurodegenerative disease in animal models of Alzheimer’s or Parkinson disease [20, 21]. A recent study has also reported an anti-inflammatory property of M30 via a down-regulation of the expression of inflammatory cytokines in a genetic model of Alzheimer’s disease [22]. Yet, there is a lack of evidence on the mechanistic effect of M30 against the oxidative stress, inflammation and neurodegeneration induced by chronic intermittent hypoxia. In this study, we hypothesized that M30 could prevent depressive behavior induced by chronic intermittent hypoxia via its antagonistic effects on the MAO-A activity and oxidative stress, resulting in inflammation, IDO-1 activation, serotonin deficiency and neurodegeneration in the rat hippocampus.

## Materials and methods

### Animal grouping and cell culture

Animal care and experimental protocol were approved and conducted according to the Committee on the Use of Live Animals in Teaching and Research (CULATR #2522–11, 3545–15), The University of Hong Kong. The Laboratory Animal Unit of the University of Hong Kong is fully accredited by the Association for Assessment and Accreditation of Laboratory Animal Care International (AAALAC international). Adult male Sprague-Dawley rats (220-250g) were put under pathogen-free condition in an air-conditioned room at constant temperature (23±1°C) provided with water and standard diet (LabDiet, 5053 (LabDiet; St. Louis, MO, USA)) ad libitum. All animals were monitored on a daily basis for body health throughout the study. The animals were divided into four experimental groups (n = 12 each), namely normoxic control (Nx), M30-treated normoxic group (Nx+M30), hypoxia-treated group (IH), M30-treated hypoxic group (IH+M30).

The SH-SY5Y cells were obtained from ATCC (Manassa, VA, USA). The cells were cultured in DMEM/F-12, supplemented with 10% fetal bovine serum, penicillin (100 U/mL), and streptomycin (100 μg/mL), which were kept in incubators with 95% air and 5% CO_2_ at 37°C.

### Intermittent hypoxic protocol and drug preparation

The normoxic control rats were kept in room air while hypoxic rats were maintained in an acrylic chamber for normobaric hypoxia in the same room. Levels of oxygen in the chamber were cycling between 21 to 5 ± 0.5% per minute (i.e. 60 hypoxic episodes per hour) for 8 hours per day diurnally for 7 consecutive days. The desired oxygen levels were established by a mixture of room air and nitrogen and monitored by an oxygen analyzer (Vacumetrics Inc., St. Ventura, CA, USA).

M30, 5[-N-Methyl-N-propargylaminomethyl]-8-hydroxyquinoline) was chemically synthesized and kindly provided by Dr. Moussa Youdim and Dr. Lin Bin. Rats were administered with a daily intraperitoneal injection of M30 (5mg/kg) 2 hours prior to the hypoxic treatment. The animals were anesthetized with halothane and then decapitated to harvest the hippocampus for experiments.

Cells were placed in an acrylic chamber in the incubator for intermittent hypoxia (repeated episodes of hypoxia at 1.5% oxygen for 4 hours followed by 21% oxygen for 4 hours) for 24 to 48 hours. MAO-A inhibitor clorgyline (10μM) or M30 (1μM) was added to the culture medium 1 hour prior to the hypoxic treatment.

### Determination of MAO-A and MAO-B activities

According to manufacturers’ instruction, hippocampi were homogenized in 50mM potassium phosphate buffer (pH = 7.4) followed by optimal dilution with the use of reaction buffer provided by Amplex Red Monoamine Oxidase Assay Kit (Invitrogen, CA, USA). Enzyme activities of MAO-A and MAO-B were determined and normalized to total protein content in each sample. The results were expressed in the percentage of control.

### Western blot

Levels of protein expression of hippocampal tissue (including whole tissue cell lysate, cytosolic or nuclear fractions) were carried out as previously described [23]. The optical density of the bands was measured and quantified by Image J (National Institute of Health, MD, USA). Primary antibodies of SOD-2, GPx-1, NFκB p65 and p50, IκBα, TNFα, IL-1β, IL-6 and COX-2 were purchased from Santa Cruz Biotechnology, CA, USA; Synapsin-1 and Synaptophysin were purchased from Novus Biologicals, USA; PSD95 and Cleaved Caspase 3 was purchased from Cell Signaling Technology; Cleaved PARP1 was purchased from Bioworld Technology; IDO-1 was purchased from antibodies-online (ABIN1714836). The data were expressed as percentage of the control.

### Enzyme-linked immunosorbent assay (ELISA)

Hippocampal serotonin (5-HT) (Enzo Life Sciences), 5-HT metabolite 5-hydroxyindoleacetic acid (5-HIAA) (Elabscience), tryptophan (TRP) (LDN Labor Diagnostika Nord GmbH & Co.KG), kynurenine (KYN) (MyBioSource) and quinolinic acid (QUIN) (Cloud Clone Corp.) were determined by ELISA quantification. The results were expressed as ratio or ng/gram wet tissue and nM respectively.

### Malondialdehyde (MDA) aAssay

Hippocampal MDA levels were examined by Bioxytech LPO-586TM kit (OxisResearch, Portland, OR). Briefly, according to manufacturer’s protocol, the reaction products were detected at 586 nm and the standard curves was constructed with 1,1,3,3-tetraethoxypropan. Protein amount of each samples were measured by Bio-Rad Protein Assay Kit (Bio-Rad, Hercules, CA). The results were normalized by protein amount. The data was expressed as μmol/mg and percentage of the control.

### GSH/GSSG ratio

Hippocampi were first homogenized in 5% metaphosphoric acid. Then, the homogenized lysate was centrifuged at 14,000g for 15min at 4°C to obtain the supernatant for glutathione determination (Enzo Life Sciences). Total amount of glutathione (GSH) and oxidized glutathione (GSSG) were determined according to manufacturer’s protocol. For reduced GSH, it was calculated as below: Reduced GSH = Total Glutathione–Oxidized GSSH.

### Golgi staining

Hippocampal CA1 and CA3 pyramidal neurons were visualized with the use of Golgi staining kit (FD Rapid Golgistain Kit (FD Neurotechnologies, MD). Dendritic spine density, length, and soma area were analyzed with the use of Neurolucida software (MicroBright-Field, USA). Soma volume was calculated with the below mathematical equation: 4/3πr^3^. Neurons chosen for analysis should be relatively separated in order to avoid the interference with neighboring impregnated neurons. Five neurons from each 100μm-thick brain section were sampled and analyzed. For the dendritic length and spine density, three to five dendrites with at least one branch point were selected for counting. Visible spines along the branch segment were counted and the spine density was expressed as number/10μm. The dendritic length, area and volume of soma were expressed as μm, μm^2^ and μm^3^ respectively.

### Behavioral tests

**Fig 1:**
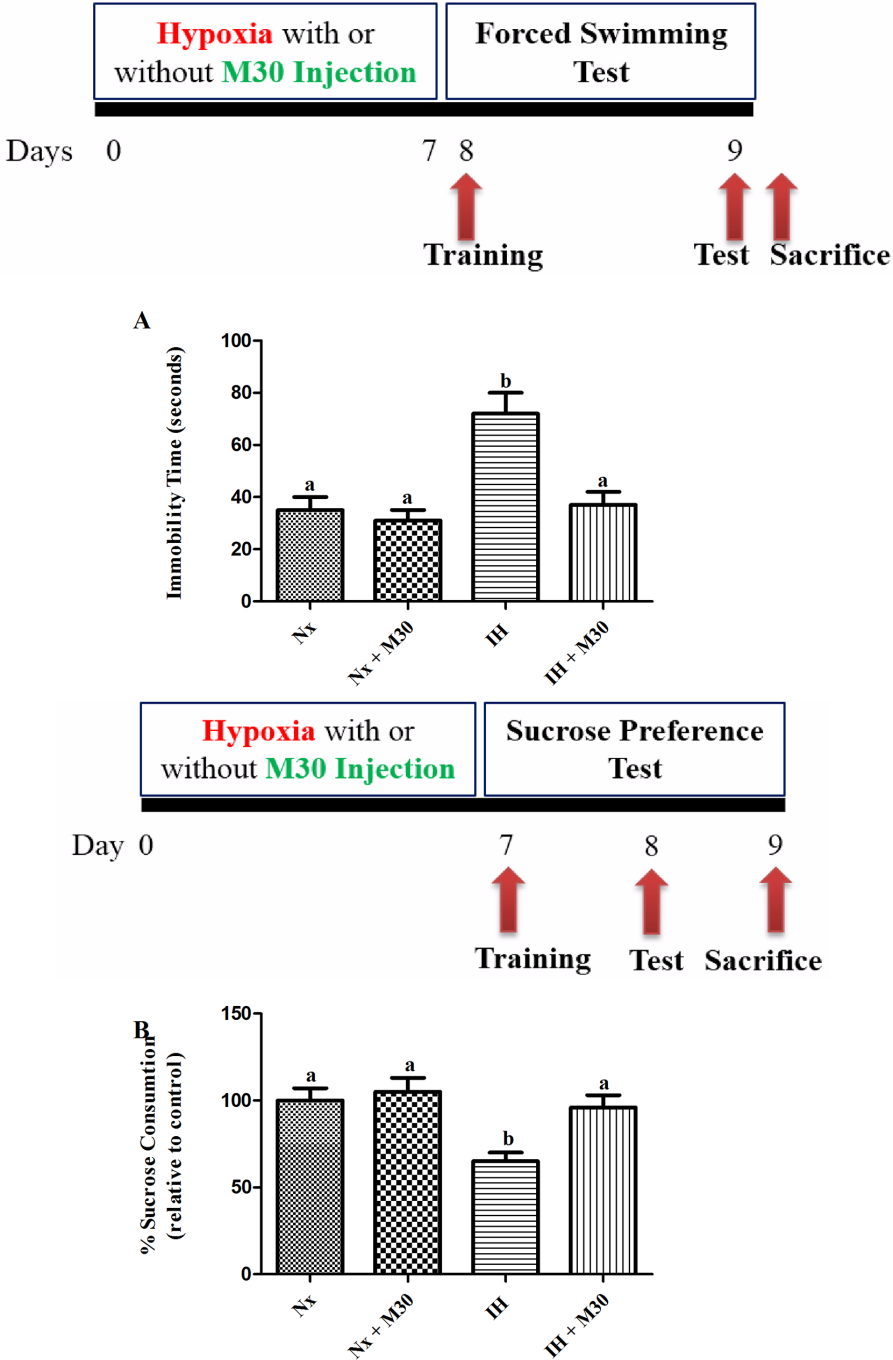
Hypoxia induced depressive-like behavior in the rat, which was significantly prevented by the M30 administration. Panels A and B represent the immobility time and percentage of sucrose consumption of the rats in normoxic (Nx), hypoxic (IH) groups; M30-treated hypoxic (IH+M30) or normoxic (Nx+M30) groups respectively. Data are mean ± SEM (n = 12). Statistical comparisons between groups were performed using the One way Anova followed by Tukey post hoc test to detect differences in all groups. A p < 0.05 was considered to be statistically significant. Different letters (e.g. a and b) mean a statistical significant change between each other.

In forced swimming test (FST), rats were put in a cylinder (60cm height X 25 cm diameter) containing tap water for a 15-min training session to learn helplessness on the following day after the hypoxic treatment (Fig 1). The animals were put into the cylinder for 5 minutes on the next day and were recorded on a video-tape for the post-analysis of the immobility time. The results were expressed in seconds. The immobility time serves as an indicator of behavioral despair, which is a main phenotype observed in rat models for depression and clinically depressed patients.

In sucrose preference test (SPT), rats were individually caged and provided the training session in which two bottles of 1% (wt/vol) sucrose solution for 24 hours on the following day after the intermittent hypoxic treatment to prevent the subtle stress when applying the sucrose consumption assessment. After that, the rats were provided one bottle of water and the other contained 1% (wt/vol) sucrose solution for 24 hours. The positions of bottles were swapped at the middle of the assessment to avoid the bias towards a particular side. No food and water deprivation was applied before the test to prevent the interference with the metabolic demands of the rats. The consumption of water and sucrose solution was recorded by weighing bottles before and after the test. Results were presented as the percentage of the sucrose solution over the total weight of liquid consumed and expressed as percentage of the control.

### Statistical analysis

Data from each group were expressed as mean ± SEM. Statistical comparisons among groups were performed using One way ANOVA followed by Tukey’s post-hoc test for multiple comparisons with the use of Graphpad Prism software (Graphpad Software Version 5.01, Inc., San Diego, USA). A p<0.05 is considered as significant difference between groups and indicated by different letter (e.g. a and b).

## Results

### Hypoxia-induced depressive behavior in rats

Behavioral despair and hedonic status of the rats were assessed by the immobility time of forced swimming test (FST) and percentage of sucrose consumption of reward-based sucrose preference test (SPT) respectively. The immobility time of hypoxic group was doubled when compared with that of the normoxic control (n = 12, Fig 1). The percentage of sucrose consumption of the hypoxic group was reduced by 35% of the control. These values were not significantly different between the M30-treated groups and the control, supporting a prophylactic effect of M30 against hypoxia-induced depressive behavior.

### Hypoxia elevated the hippocampal MAO-A activity

**Fig 2:**
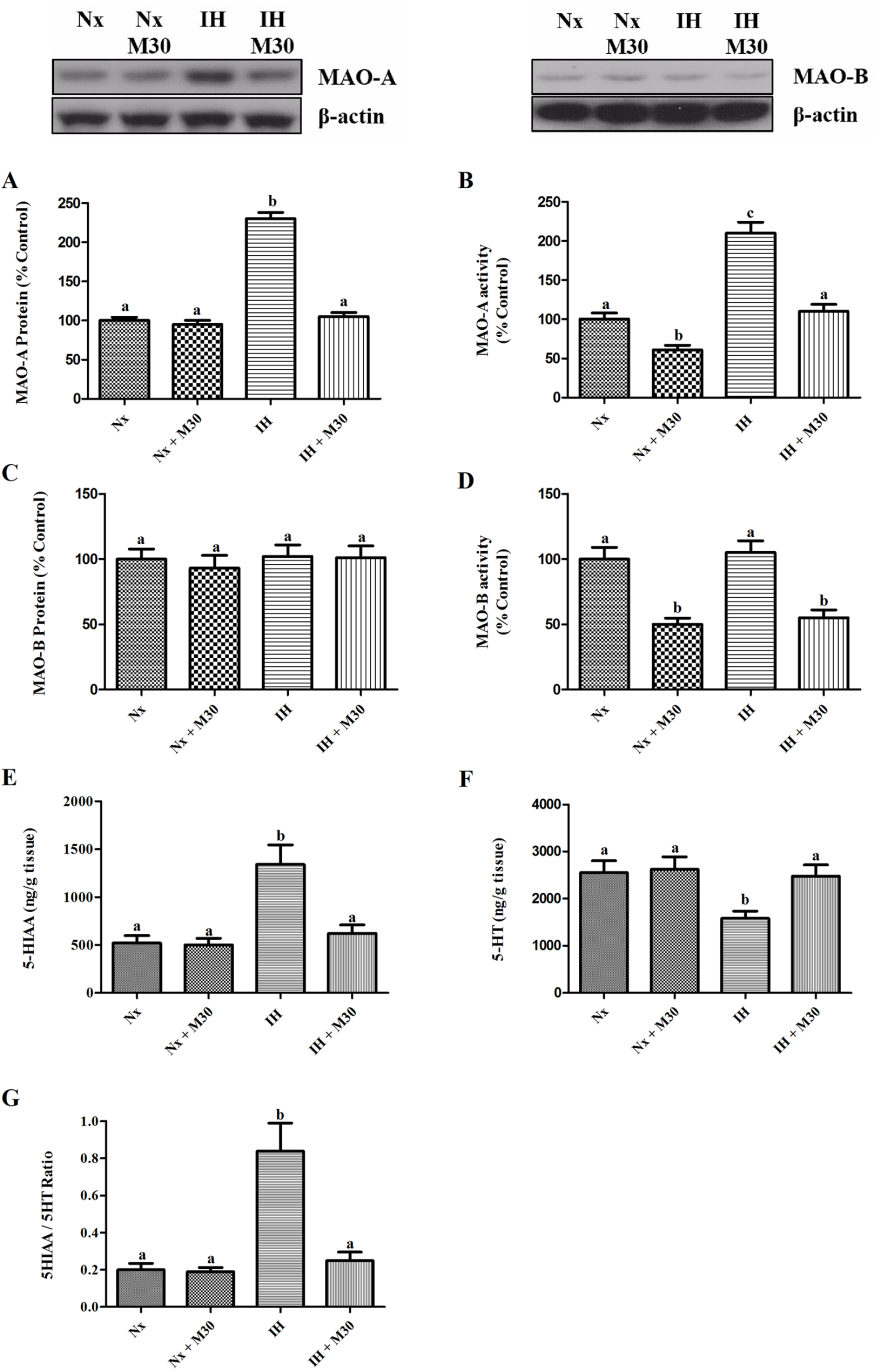
Hypoxia increased the metabolic turnover of serotonin mediated by the elevated activity of MAO-A but not MAO-B in the hippocampus. Serotonin (5-HT) level was also lowered in the hypoxic group. These alterations were significantly attenuated by M30. Levels of protein expression of (A) MAO-A, (B) MAO-B, activity of (C) MAO-A, (D) MAO-B, (E) 5-HT, (F) 5-HIAA and (G) 5-HIAA/5-HT are summarized. β-actin was the internal control. Data are presented as Mean ± SEM (n = 8). Statistical comparisons between groups were performed using the One way Anova followed by Tukey post hoc test to detect differences in all groups. A p < 0.05 was considered to be statistically significant. Different letters (e.g. a and b) mean a statistical significant change between each other.

Levels of the expression and activity of MAO-A in the hypoxic group were significantly elevated by two folds of the control (n = 8, Fig 2), but there were no changes in the expression and activity of MAO-B. Also, the 5-HIAA/5-HT ratio was markedly increased in the hypoxic group (n = 8, Fig 2). No significant differences were found between the M30-treated hypoxic group and the control. Of note, MAO-A and MAO-B activities were lowered by half in the M30-treated groups. Results suggest that the upregulation of MAO-A induced by hypoxia resulted in a deregulated serotonin metabolism, which was neutralized by M30.

### Neuroarchitectural alterations induced by hypoxia

**Fig 3:**
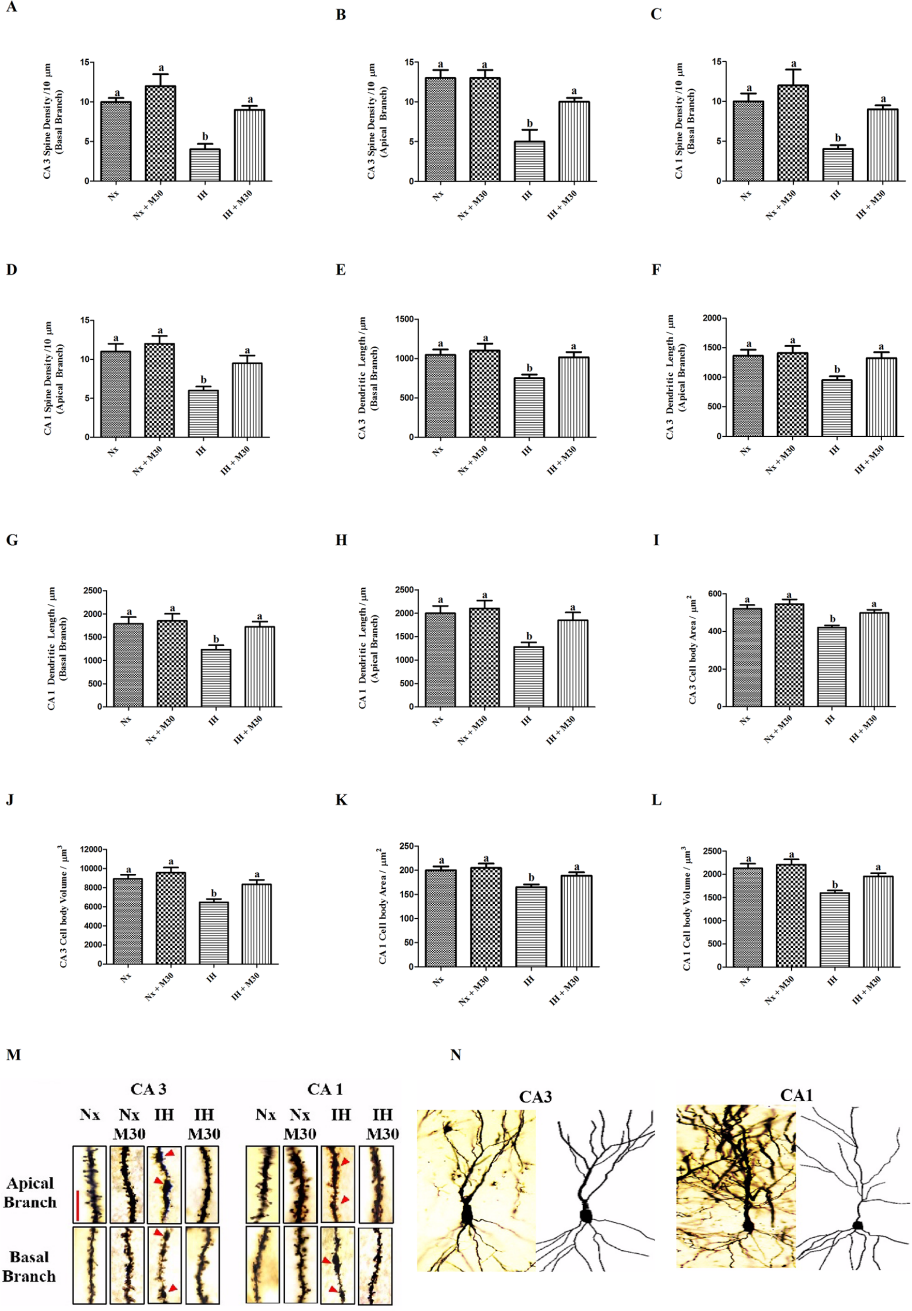
Hypoxia induced decreases in dendritic spine density, length, soma size and volume of the apical and basal branches of CA1 and CA3 pyramidal neurons. Levels of the dendritic spine densities of (A) CA3 basal branch, (B) CA3 apical branch, (E) CA1 basal branch, (F) CA1 apical branch; Levels of the dendritic length of (C) CA3 basal branch, (D) CA3 apical branch, (G) CA1 basal branch, (H) CA1 apical branch; Levels of the cell body area (I) CA3 pyramidal neuron, (J) CA1 pyramidal neuron; Levels of the cell body volume (K) CA3 pyramidal neuron, (L) CA1 pyramidal neuron in the hippocampus of the normoxic (Nx) or hypoxic (IH) groups; M30-treated hypoxic (IH+M30) or normoxic (Nx+M30) groups are summarized. Dendritic varicosity formations responsible for reduction in dendritic spine density were indicated by the arrows. Panel M summarized representative photomicrographs of apical and basal dendrites of both CA3 and CA1 pyramidal neurons showing dendritic spines. Magnification:100X, Scale bar = 10μm. Panel N illustrated Camera Lucida drawing of representative CA3 and CA1 pyramidal neurons. Data are presented as Mean ± SEM (n = 8). Statistical comparisons between groups were performed using the One way Anova followed by Tukey post hoc test to detect differences in all groups. A p < 0.05 was considered to be statistically significant. Different letters (e.g. a and b) mean a statistical significant change between each other.

Golgi staining was performed to evaluate dendritic structural changes of hippocampal pyramidal CA1 and CA3 neurons. We observed dendritic abnormalities of both apical and basal branches of neurons shown by reduced dendritic spine densities and length and also the area and volume of somata in the hypoxic group (n = 12, Fig 3). Also, a significant increase in the dendritic varicosity formations was observed in the hypoxic group. Administration of M30 effectively ameliorated neurodegeneration induced by hypoxia and no observable aberrant alterations were found in the M30-treated groups.

### Inhibition of MAO-A by M30 mitigated hypoxia-induced oxidative stress

**Fig 4:**
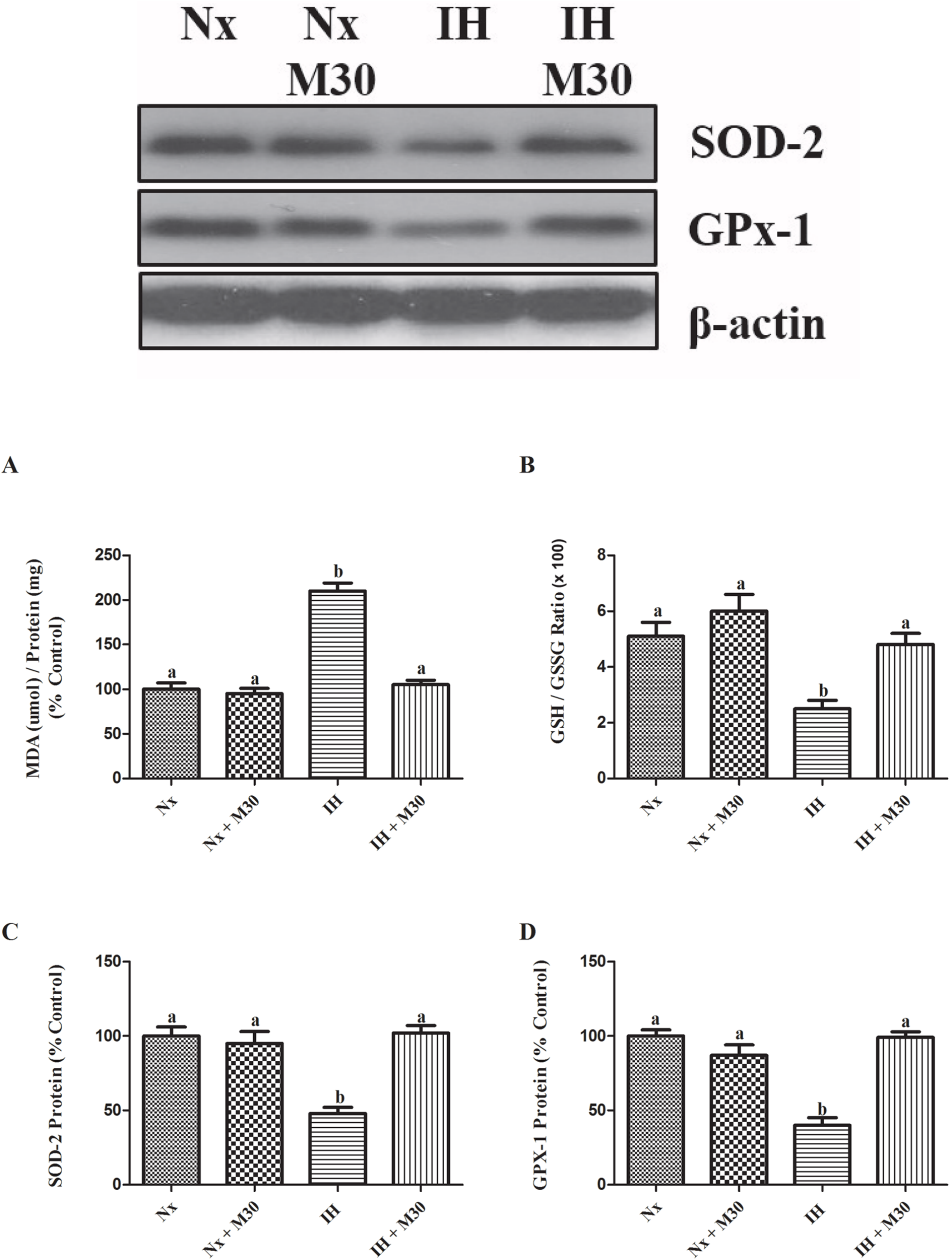
Administration of M30 attenuated oxidative stress induced by hypoxia. Levels of (A) MDA content, (B) GSH/GSSG ratio and the protein expression of (C) SOD-2 and (D) GPx-1 and in the hippocampus of the normoxic (Nx) or hypoxic (IH) groups; M30-treated hypoxic (IH+M30) or normoxic (Nx+M30) groups are summarized. β-actin was an internal control. Data are mean ± SEM (n = 8). Statistical comparisons between groups were performed using the One way Anova followed by Tukey post hoc test to detect differences in all groups. A p < 0.05 was considered to be statistically significant. Different letters (e.g. a and b) mean a statistical significant change between each other.

Hydrogen peroxide is one of the products of deamination by MAO-A, which is a source of reactive oxygen species (ROS) leading to oxidative stress. The level of MDA, a lipid peroxidation marker, was significantly elevated by two folds in the hypoxic group (n = 8, Fig 4). Also, there was a significant decrease in the GSH/GSSG ratio (n = 8, Fig 4). In addition, levels of protein expressions of antioxidant enzymes SOD-2 and GPx-1 were lowered, respectively, by 50% and 60% in the hypoxic group when compared to the control (n = 8, Fig 4). There were no significant differences between the M30-treated groups and the control.

### Hypoxia induced redox-sensitive NFκB canonical pathway-dependent inflammation

**Fig 5:**
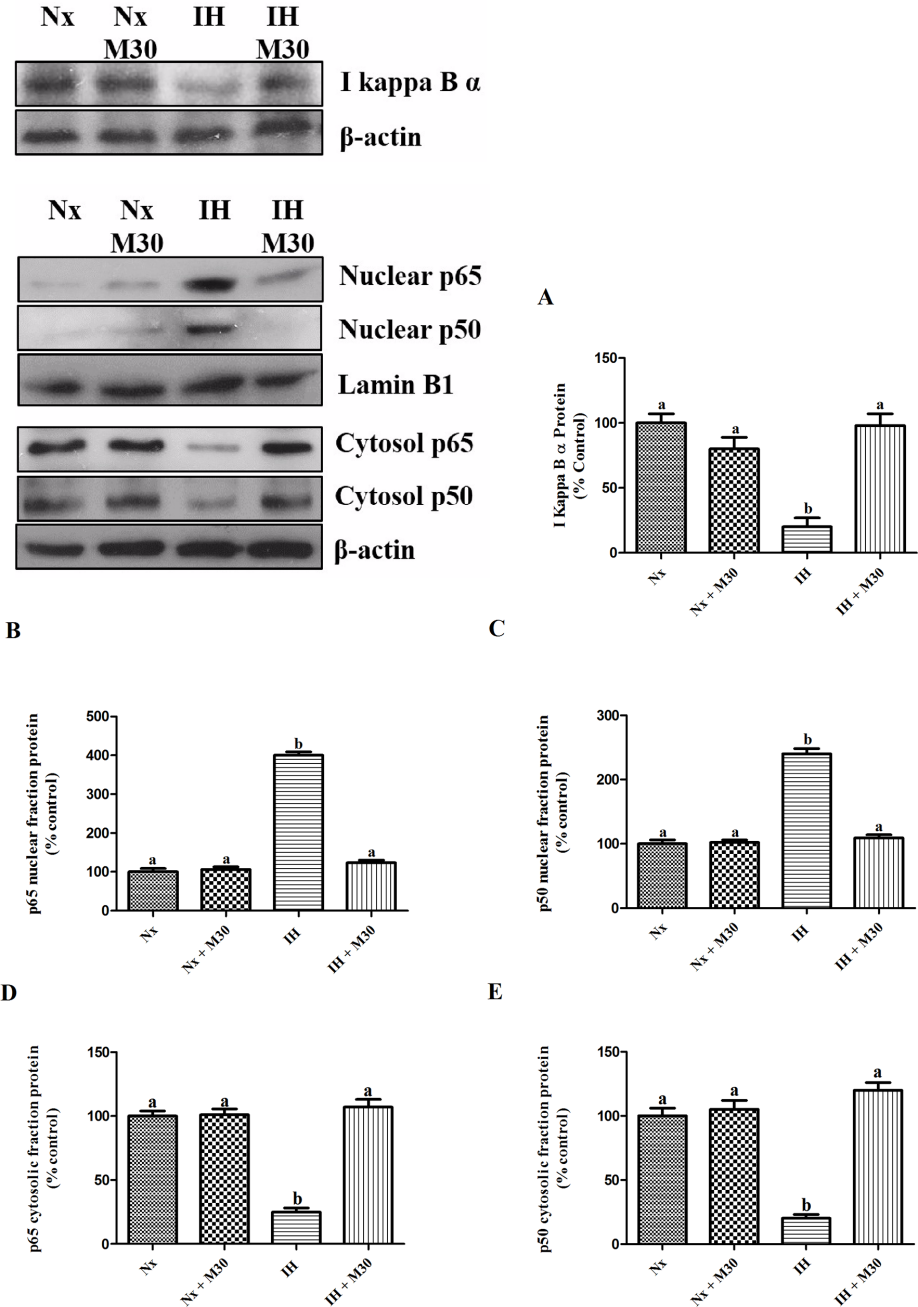
Hypoxia induced the degradation of IκBα in the redox-sensitive NFκB canonical pathway and the nucleus translocation of NFκB member p65 and p50. Levels of (A) IκBα, nuclear protein expression of (B) p65, (C) p50, cytosolic protein expression of (D) p65, (E) in the hippocampus of the normoxic (Nx) or hypoxic (IH) groups; M30-treated hypoxic (IH+M30) or normoxic (Nx+M30) groups are summarized. Lamin B1 and β-actin were an internal control of the nuclear fraction and cytosolic fraction respectively. Data are mean ± SEM (n = 8). Statistical comparisons between groups were performed using the One way Anova followed by Tukey post hoc test to detect differences in all groups. A p < 0.05 was considered to be statistically significant. Different letters (e.g. a and b) mean a statistical significant change between each other.

**Fig 6:**
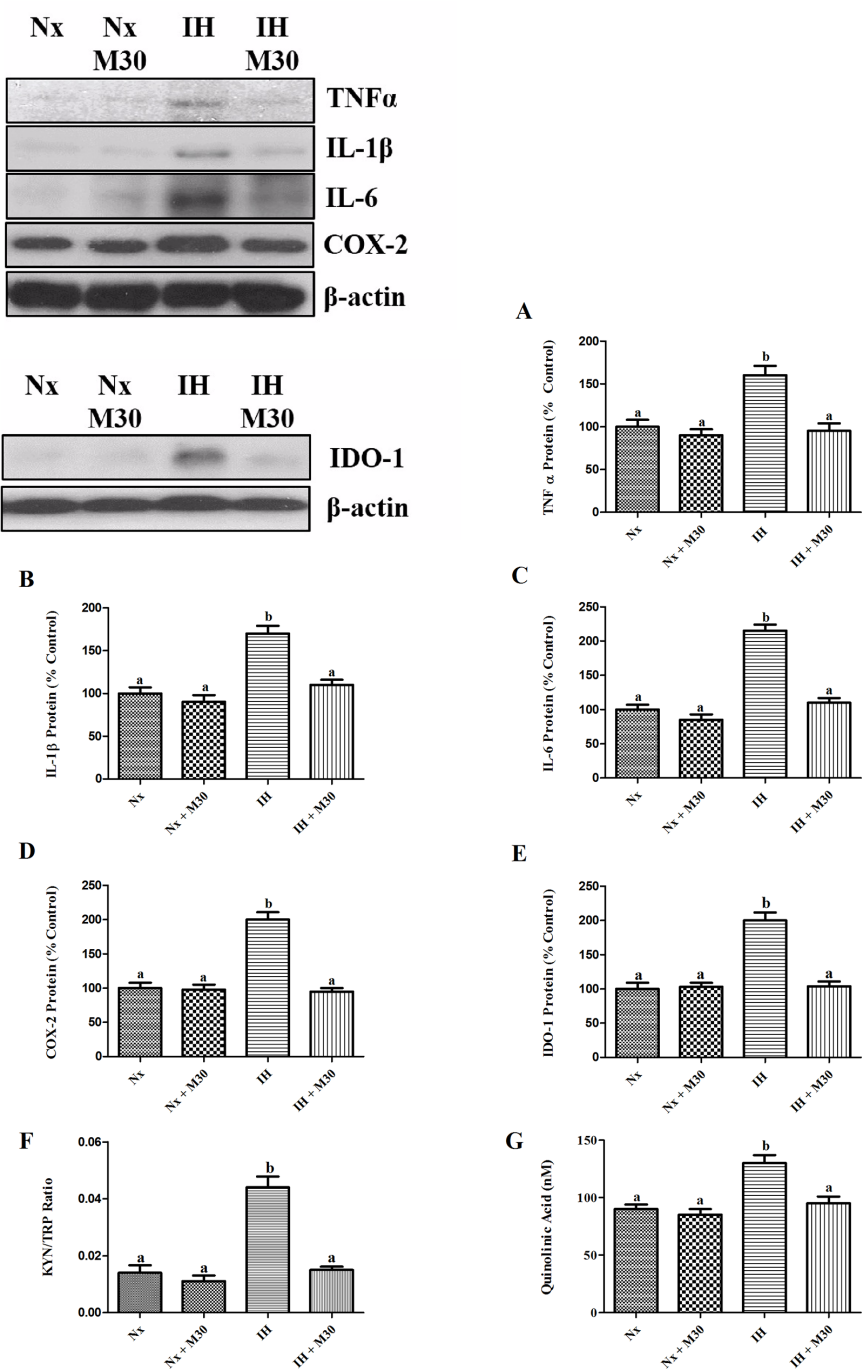
Hypoxia induced inflammation and activated cytokine-responsive IDO-1 in the hippocampus. Levels of the protein expression (A) TNFα, (B) IL-1β, (C) IL-6, (D) COX-2, (E) IDO-1, (F) the ratio of KYN/TRP (IDO-1 activity) and (G) the level of QUIN are summarized. Data are presented as Mean ± SEM (n = 8). Statistical comparisons between groups were performed using the One way Anova followed by Tukey post hoc test to detect differences in all groups. A p < 0.05 was considered to be statistically significant. Different letters (e.g. a and b) mean a statistical significant change between each other.

The level of IκBα was significantly decreased by 80% in the hypoxic group when compared to the control. Also, levels of NFκB p65 and p50 in the hypoxic group were significantly reduced in the cytosolic fraction and were markedly increased in the nuclear fraction (n = 8, Fig 5). Besides, there were significant increases in the protein expression of inflammatory cytokines TNFα, IL-1β, IL-6 and COX-2 by about two folds in the hypoxic group when compared to the control (n = 8, Fig 6). M30 treatment normalized the IκBα degradation, nuclear translocation of NFκB and cytokine expression to the control level.

### The expression and activity of IDO-1 activated by hypoxia

IDO-1 activity were evaluated by the ratio changes of its substrate tryptophan and enzymatic metabolite kynurenine. The expression level of IDO-1 and the ratio of kynurenine to tryptophan were significantly increased, respectively, by two folds and four folds in the hypoxic group (n = 8, Fig 6). Consistently, the level of downstream IDO-1 metabolite QUIN was also increased by hypoxia. M30 treatment mitigated the elevated level of IDO-1 expression and activity, and the increased level of QUIN.

### Hypoxia induced losses of synaptic proteins and neuronal apoptosis

**Fig 7:**
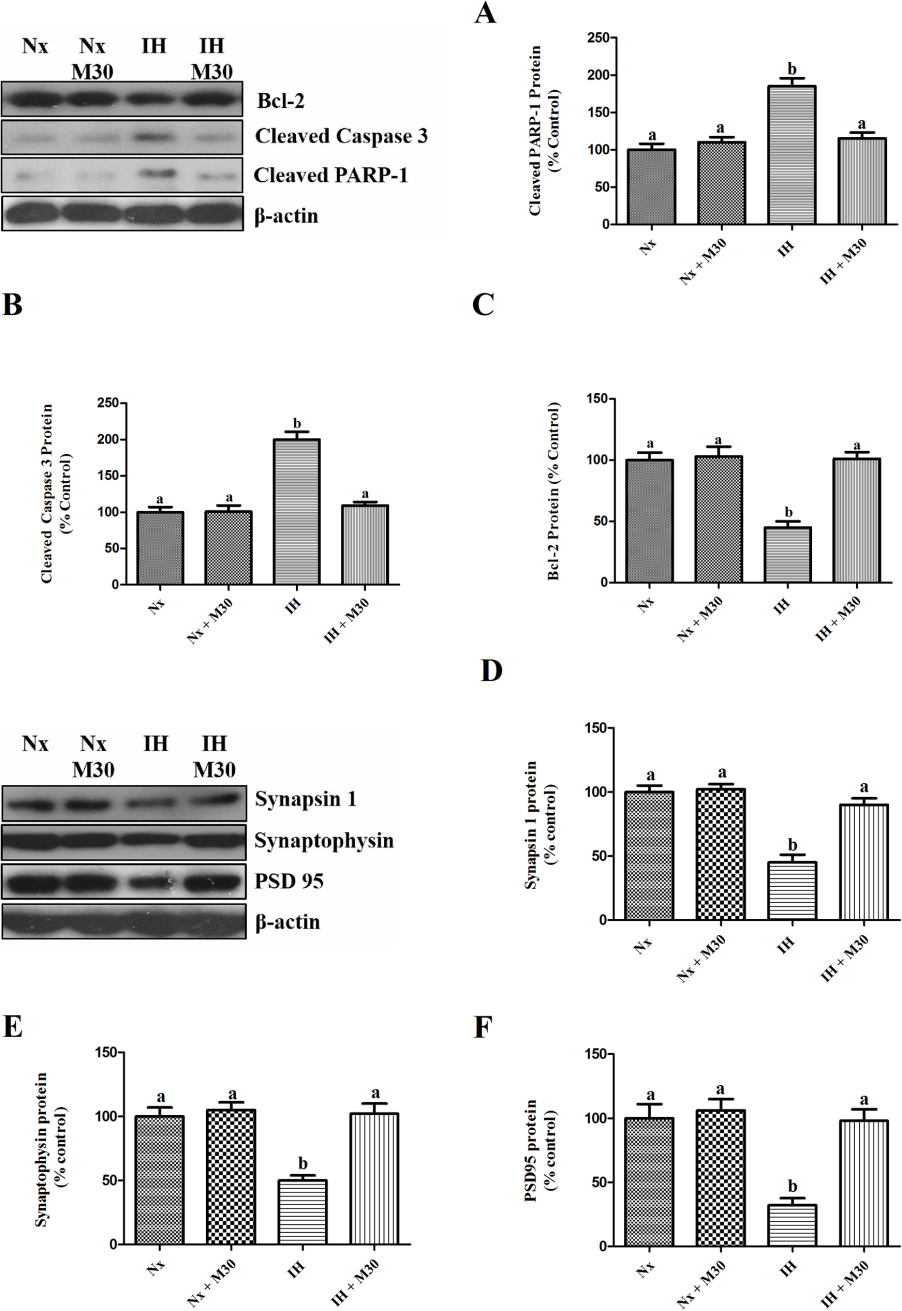
Hypoxia induced neuronal apoptosis and losses of pre-synaptic vesicle proteins and post-synaptic. Levels of protein expression of (A) Bcl-2, (B) Cleaved Caspase 3, (C) Cleaved PARP-1, (D) Synapsin-1, (E) Synaptophysin and (F) PSD-95 in the hippocampus of the normoxic (Nx) or hypoxic (IH) groups; M30-treated hypoxic (IH+M30) or normoxic (Nx+M30) groups are summarized. β-actin was an internal control. Data are presented as Mean ± SEM (n = 8). Statistical comparisons between groups were performed using the One way Anova followed by Tukey post hoc test to detect differences in all groups. A p < 0.05 was considered to be statistically significant. Different letters (e.g. a and b) mean a statistical significant change between each other.

There were dramatic decreases in the expression of pre-synaptic proteins synapsin-1 and synaptophysin, and post-synaptic protein PSD95 in the hypoxic group, respectively, by 55%, 50% and 68% of the control (n = 8, Fig 7). Furthermore, the expression of anti-apoptotic protein Bcl-2 was much lowered by 55% of the control and the expression of apoptotic markers cleaved caspase 3 and cleaved PARP-1 was doubled in the hypoxic group (n = 8, Fig 7). Administration of M30 effectively ameliorated losses of synaptic proteins and neuronal apoptosis induced by hypoxia.

### Hypoxia increased MAO-A expression in SH-SY5Y cells

**Fig 8:**
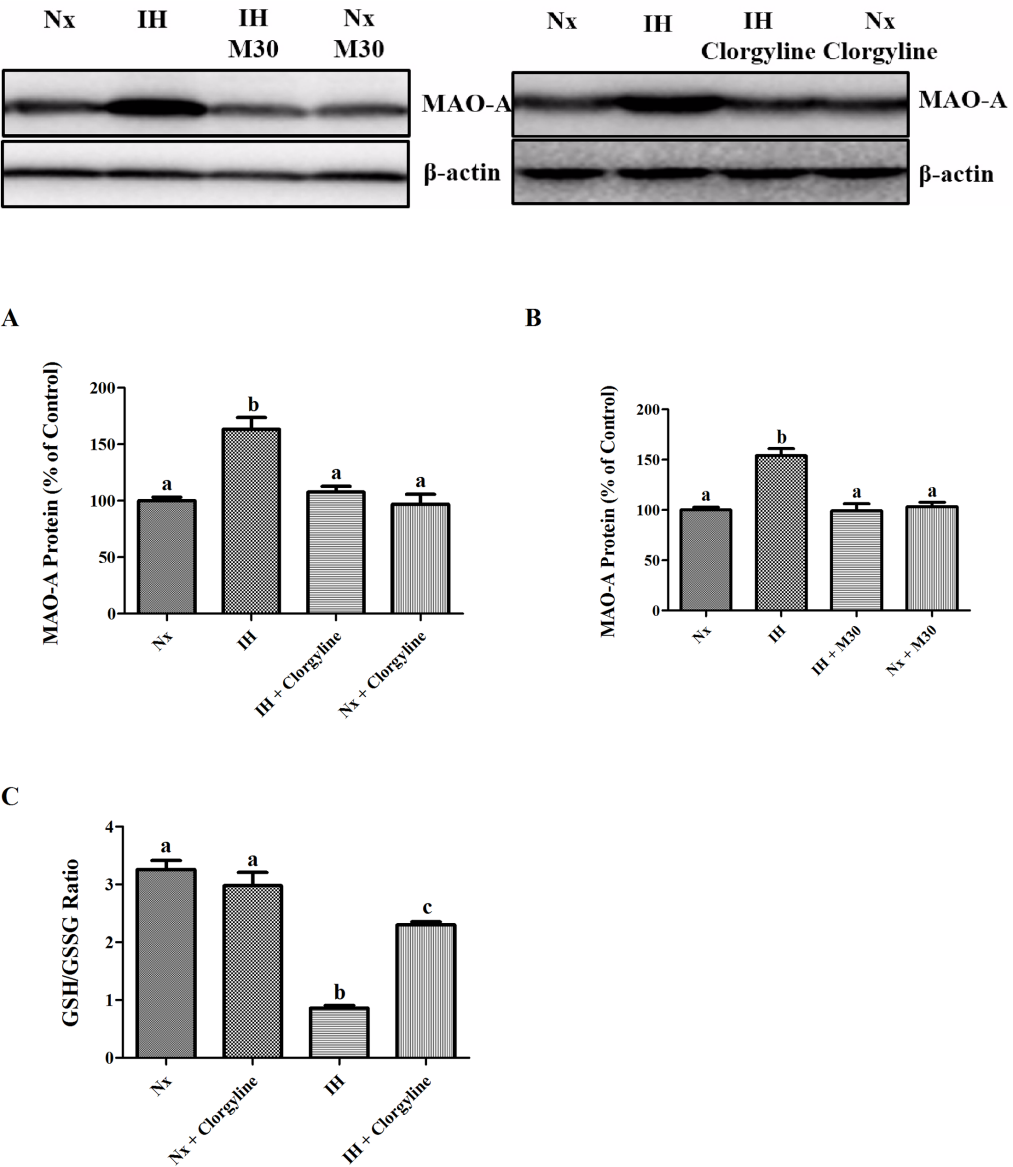
The level of protein expression of MAO-A was increased by exposure to intermittent hypoxia (IH) in SH-SY5Y cells, which was antagonized by M30 at 1μM or clorgyline at 10μM (Panel A and B). Hypoxia significantly reduced the GSH/GSSG ratio in SH-SY5Y cells, which was partially attenuated by clorgyline at 10μM (Panel C). Data are mean ± SEM (n = 6). Statistical comparisons between groups were performed using the One way Anova followed by Tukey post hoc test to detect differences in all groups. A p < 0.05 was considered to be statistically significant. Different letters (e.g. a and b) mean a statistical significant change between each other.

In human SH-SY5Y cells expressing MAO-A but not MAO-B, the level of protein expression of MAO-A was significantly increased by 50% following the hypoxic treatment (Fig 8). The level of MAO-A expression was normalized by the pretreatment of the cells with M30 or clorgyline. Hypoxia also significantly reduced the GSH to GSSG ratio, which was partially normalized by clorgyline (Fig 8).

## Discussion

This is the first report to delineate a pathophysiological role of the anomalous MAO-A activity upregulated by chronic intermittent hypoxia in oxidative stress and inflammation, which significantly activates the IDO-1 activity and contributes to serotonin deficiency and neurodegeneration closely associated with the clinical manifestation of the brain of patients with depression. This may help to explain high rates of depression in both community (17%) and clinical (21–41%) populations with obstructive sleep apnea (OSA) [2, 4, 24–26]. Our results are clinically relevant in its proposed pathophysiological causality between OSA and depression. Yet, we are aware of the limitation of the rodent model with the hypoxic treatment in an attempt to link degenerative neuropathies to the arterial oxygen desaturation simulating the pathophysiological consequences in a severe OSA condition, which is an important parameter in the clinical assessment. Intriguingly, we demonstrated the prophylactic effect of the synthetic compound M30 against the aberrant changes in the hippocampus and depressive behavior, which is indicative of targeting the MAO-A activity, oxidative stress and inflammation to retard the neurodegeneration induced by hypoxia. The administrative dose of M30 has been reportedly safe for the experimental usage and did not cause any noticeable physiological or histopathological changes in the healthy animals [27].

A major finding of this study is that MAO-A but not the MAO-B activity was up-regulated in the hippocampus of hypoxic rats, which is in consistent with the increased MAO-A expression. This could cause an increased catabolic deamination of 5-HT resulting in increases in the level of the metabolite 5-HIAA and its ratio to 5-HT, leading to a lowered serotonin availability that were reportedly shown in the brain of clinically depressed patients and animal models of depression [28, 29]. These findings are also in agreement with studies reporting that MAO-A inhibitors could significantly improve depressive symptoms in the patient and rodent models [30–32]. Importantly, our results provide novel evidence to link the upregulation of MAO-A to neurodegeneration as a pathophysiological consequence of chronic intermittent hypoxia. We also demonstrated evidence for a proof of concept that the daily M30 administration significantly antagonized the MAO-A upregulation and consequently prevented the depressive behavior induced by hypoxia. Blockade of MAO-A activity could be a therapeutic approach to preventing the neurodegeneration in patients with severe conditions of OSA that is known to increase the risk for depression.

Oxidative stress is a significant adverse consequence of chronic intermittent hypoxia due to an increased production of reactive oxygen species (ROS). In this context, the upregulated MAO-A activity could result in an increased enzymatic production of hydrogen peroxide as a by-product of the catalytic deamination of monoamines. In effect, elevated levels of ROS deplete endogenous GSH antioxidant capacity and cause the lipid peroxidation of membrane forming MDA. Indeed, our results showed an elevated level of oxidative stress in the hippocampus of hypoxic rats with significant decreases in the GSH to GSSG ratio and increases in the MDA level. Also, the protein expression of antioxidant enzymes SOD-2 and GPx-2 was significantly lowered. These changes were markedly neutralized by the M30 treatment, strongly supporting that the chemical properties of M30, namely for the MAO inhibition and scavenging ROS, were effective to antagonize the pathophysiological consequences induced by chronic intermittent hypoxia. M30 is also highly permeable to the blood brain barrier and selective to the brain MAO-A activity [33], which is potentially an advantage to limit the non-specific side effects of MAO inhibitors.

Inflammation has been proposed as a prominent factor in the induction of depressive symptoms, which could be mediated by the activation of cytokine-responsive IDO-1. Studies have also shown that neuroinflammation was closely linked to oxidative stress via redox sensitive NFκB canonical pathway, forming a vicious cycle in the production of ROS and inflammatory cytokines, causing tissue damages [23, 34]. Indeed, we found significant increases in the degradation of IκBα and the translocation of NFκB p65 and p50 from the cytosol to nucleus. Consequently, the activation of NFκB canonical pathway results in the elevated expression of inflammatory cytokines TNFα, IL-1β, IL-6 and COX-2. Intriguingly, the M30 treatment remarkably mitigated the activation of redox-sensitive NFκB cascade and the expression of cytokines, which is consistent with an anti-inflammatory property of M30 reported recently [22].

The augmented IDO-1 activity has been proposed to play a pathogenic role in the catabolic conversion of tryptophan to kynurenine, causing a decrease in tryptophan availability for the 5-HT biosynthesis leading to serotonin deficiency [35, 36]. Our results showed a significant increase in the IDO-1 expression and activity in parallel with a significantly lowered 5-HT level in the hippocampus of hypoxic rats. These were in consistent with findings of previous reports showing 5-HT depletion as a result of elevated IDO-1 activities could be restored by the administration of anti-inflammatory agents [37, 38]. In fact, we showed that M30 significantly decreased MAO-A activity and oxidative stress, which could lessen the activation of redox-sensitive NFκB cascade and the expression of cytokines that elevate the cytokine responsive IDO-1 activity in the hippocampus of hypoxic rats.

Neurodegeneration is an important neuropathic feature of depression observed in both patients and animal models [39, 40], which is closely associated with and attributed to apoptosis as a result of oxidative stress and inflammation leading to the activation of intrinsic and extrinsic cascades [23, 41]. These are illustrated in our results showing significant increases in the level of apoptotic markers cleaved caspase 3 and cleaved PARP 1, and marked decreases in the level of anti-apoptotic protein Bcl-2. By targeting MAO-A upregulation, oxidative stress and inflammation, M30 could abrogated neuronal apoptosis induced by hypoxia, which is in line with previous reports illustrating anti-apoptotic property of M30 in the animal models of Parkinson’s diseases and stress-induced depression [20, 42].

Losses of synaptic proteins were observed in the brains of patients and animals with depression and were proposed to be closely related to the onset of depression [43, 44]. We found that there were significant down-regulation of expressions of pre-synaptic vesicular proteins synapsin-1 and synaptophysin, and post-synaptic protein PSD95 in the hippocampus of hypoxic rats, which were remarkably ameliorated by the M30 treatment. This is in agreement with the observation that the degradation of synaptic proteins was mediated by apoptotic caspase enzymes [45]. These results give strong support to the contention that M30 confers protective effects against synaptic degeneration induced by hypoxia.

Golgi staining vividly revealed adverse effects on the neuroarchitecture induced by hypoxia. There were remarkable reductions in the dendritic spine density and dendritic length on both apical and basal branches of the CA1 and CA3 pyramidal neurons in the hippocampus of hypoxic rats. It has been reported that hypoxia could induce NMDA overactivation that leads to excitotoxicity and causes ultrastructural damages in the hippocampus [46]. In fact, NMDA excitotoxicity is a major cause of varicosities formation in the dendritic spines and decreases the spine density [47, 48]. Particularly, the catabolic degradation of tryptophan by IDO-1 produces neurotoxins such as quinolinic acid that agonistically effect on the activation of NMDA receptors and potentially cause the formation of varicosities when IDO-1 activity is augmented under hypoxic conditions. The area and volume of the soma were markedly decreased in the hippocampus of hypoxic rats, which are in line with the pathogenic processes of DNA fragmentation and condensation in neuronal apoptosis [49]. These defective changes in the spines and somata were not found in the rat treated with M30, strongly supporting a pathogenic role of MAO-A in oxidative stress and inflammation leading to IDO-1 activation that causes the neurodegeneration. Results are also in consistent with the observation that MAO-A inhibitors could rescue the abnormal dendritic structure upon exposure to chronic stress [50].

Finally, the effect of hypoxia on the MAO-A upregulation was confirmed by the findings in cultured human SH-SY5Y cells, which constitutively express MAO-A but not MAO-B. The MAO-A expression was significantly upregulated in the hypoxic group, providing evidence to argue a non-specific effect of hypoxia on the MAO-A upregulation. The MAO-A upregulation was blocked by M30 or clorgyline, suggesting a crucial role of MAO-A in the pathophysiological cascade initiated by chronic intermittent hypoxia, which drives the adverse consequences towards neurodegeneration. This is summarized in S1 Fig. illustrating the pathophysiological mechanism underlying the onset of neurodegeneration and depressive behavior induced by chronic intermittent hypoxia, which also highlights the effect of M30 targeting the MAO-A that significantly contribute to oxidative stress and inflammation leading to serotonin deficiency and neurodegeneration.

## Conclusions

Chronic intermittent hypoxia upregulates the expression and activity of MAO-A resulting in oxidative stress, inflammation, activation of IDO-1, causing serotonin deficiency and neurodegeneration, which could be potently antagonized by M30 against the upregulation of MAO-A activity and the production of free radicals. MAO-A is an indicative drug target to prevent the onset of depressive symptoms in OSA patients.

## Supporting information

S1 Fig A schematic summary of the pathophysiological cascade induced by chronic intermittent hypoxia (CIH) leading to depression. (PDF)
