# Supplementary figures and images for: Monoamine oxidase A upregulated by chronic intermittent hypoxia activates indoleamine 2,3-dioxygenase and neurodegeneration
Source: PLoS One. 2017 Jun 9;12(6):e0177940. doi: 10.1371/journal.pone.0177940 (PMC5466431; doi:10.1371/journal.pone.0177940)

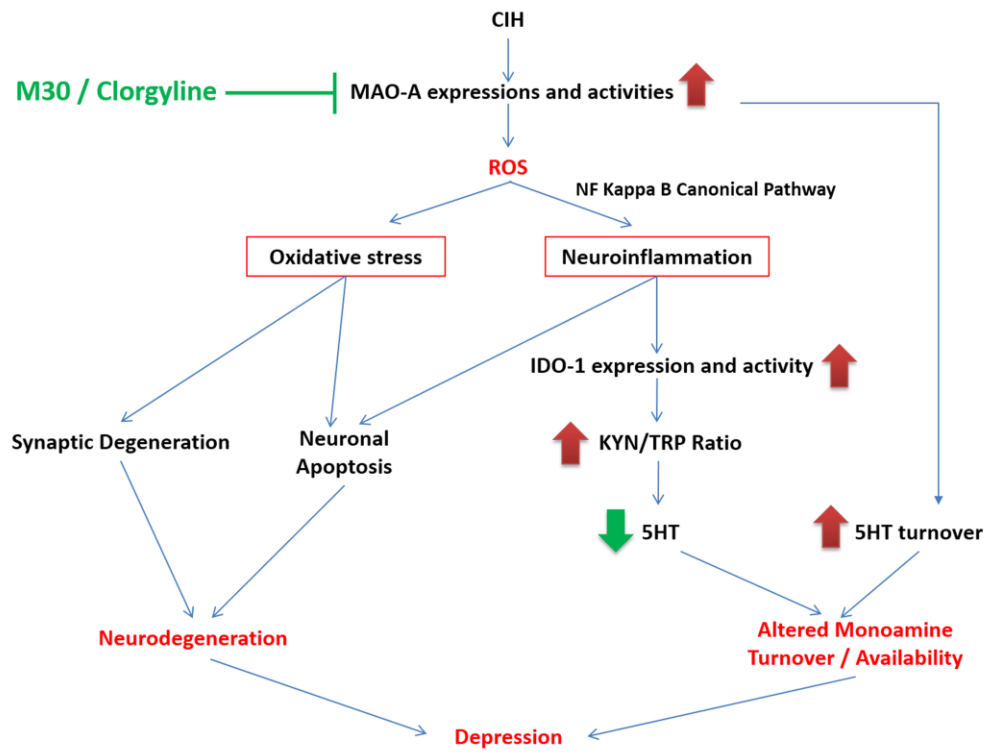

Supplement: S1 Fig — (PDF) [file pone.0177940.s001.pdf]
